# Bridging the Gap to Non-toxic Fungal Control: Lupinus-Derived Blad-Containing Oligomer as a Novel Candidate to Combat Human Pathogenic Fungi

**DOI:** 10.3389/fmicb.2017.01182

**Published:** 2017-06-28

**Authors:** Ana M. Pinheiro, Alexandra Carreira, Thomas A. K. Prescott, Ricardo B. Ferreira, Sara A. Monteiro

**Affiliations:** ^1^Linking Landscape, Environment, Agriculture and Food, Instituto Superior de Agronomia, Universidade de LisboaLisboa, Portugal; ^2^CEV, SA, Parque Industrial de Cantanhede/Biocant-ParkCantanhede, Portugal; ^3^Royal Botanic Gardens, KewRichmond, United Kingdom

**Keywords:** Blad-containing oligomer, antifungal, metal chelation, metal homeostasis, multitarget mode of action, toxicology

## Abstract

The lack of antifungal drugs with novel modes of action reaching the clinic is a serious concern. Recently a novel antifungal protein referred to as Blad-containing oligomer (BCO) has received regulatory approval as an agricultural antifungal agent. Interestingly its spectrum of antifungal activity includes human pathogens such as *Candida albicans*, however, its mode of action has yet to be elucidated. Here we demonstrate that BCO exerts its antifungal activity through inhibition of metal ion homeostasis which results in apoptotic cell death in *C. albicans*. HIP HOP profiling in *Saccharomyces cerevisiae* using a panel of signature strains that are characteristic for common modes of action identified hypersensitivity in yeast lacking the iron-dependent transcription factor Aft1 suggesting restricted iron uptake as a mode of action. Furthermore, global transcriptome profiling in *C. albicans* also identified disruption of metal ion homeostasis as a potential mode of action. Experiments were carried out to assess the effect of divalent metal ions on the antifungal activity of BCO revealing that BCO activity is antagonized by metal ions such as Mn^2+^, Zn^2+^, and Fe^2+^. The transcriptome profile also implicated sterol synthesis as a possible secondary mode of action which was subsequently confirmed in sterol synthesis assays in *C. albicans*. Animal models for toxicity showed that BCO is generally well tolerated and presents a promising safety profile as a topical applied agent. Given its potent broad spectrum antifungal activity and novel multitarget mode of action, we propose BCO as a promising new antifungal agent for the topical treatment of fungal infections.

## Introduction

Relative to antibacterial drug discovery, antifungal drug research has received less attention, (Delarze and Sanglard, 2015; Ngo et al., 2016); this is despite the fact that 1.2 billion people worldwide suffer from fungal diseases (Denning and Bromley, 2015) and mortality rates may exceed those caused by tuberculosis or malaria (Brown et al., 2012). Unlike bacterial infections, which can be treated aggressively and with few toxic side effects, the shared eukaryotic nature of fungi and mammals presents a greater challenge when seeking selective low toxicity candidate molecules (Geiser, 2015). The pace of developing new antifungal drugs has been extremely slow, and with numerous failures (Liu et al., 2016). Most new promising compounds end up failing during the final development stages, mostly because of their mode of action (promoting fungal resistance) and/or toxicity issues. This low success rate has definitely discouraged the pharmaceutical industry from investing their resources on this type of research and explains the absence of new drug classes since echinocandins were introduced in 2001.

A restricted number of chemical classes are currently in clinical use as antifungal agents (Delarze and Sanglard, 2015) and novel resistance profiles to these drugs are frequently reported (Papon et al., 2007; Perlin, 2007; Grossman et al., 2015; Sanguinetti et al., 2015; Kołaczkowska and Kołaczkowski, 2016; Morschhäuser, 2016), including development of multi-drug resistance strains (Sanglard, 2016). Only minimal resistance to the polyene amphotericin B (AMB) has evolved despite more than 30 years of intensive clinical use (Kanafani and Perfect, 2008; Davis et al., 2015), probably due to its multitarget mode of action (Gray et al., 2012). However AMB is well known for its side effects and toxicity (Spampinato and Leonardi, 2013; Nett and Andes, 2016). A novel class of antifungal drugs, named orotomides, is now emerging, with very promising results in dimorphic and filamentous fungi, particularly for *Aspergillus* spp., and is currently in late phase 1 clinical trials for the treatment of invasive aspergillosis (Oliver et al., 2016). Nonetheless, it acts via a novel mechanism of action that targets a single enzyme (inhibition of the dihydroorotate dehydrogenase), and it has no activity against human pathogenic yeasts (Oliver et al., 2016). Generally, drugs acting on a single cellular target are more likely to encounter the problem of drug resistance (Wong et al., 2014).

Recently, we reported the discovery of an edible polypeptide, named Blad, which accumulates in *Lupinus* cotyledons (Monteiro et al., 2015) and occurs naturally as part of a 210 kDa oligomer (BCO, after Blad-containing oligomer). BCO exhibits potent and broad-spectrum fungicidal activity, comparing favourably to fluconazole (FLC) and AMB in *in vitro* (Pinheiro et al., 2016) and surpassing some commercially available fungicides in greenhouse and field conditions. BCO is currently on sale in the United States, Canada, and South Korea under the trade name Fracture^TM^. Other certification processes are currently under way worldwide. The highly complex and multitarget mechanism of action of BCO (Pinheiro et al., 2016) was already aknowledged by the Fungicide Resistance Action Committee (FRAC), with its inclusion in a new mode of action category, M12, on the 2016 issue of the FRAC Code List© for agricultural applications.

With such a wide range of bioactivities, anyone skilled-in-the-art would expect BCO to be toxic to mammal cells as well. In this study, we demonstrate that this does not seem to be the case. In addition to its inherent capacity to bind (lectin activity) and cleave chitin (*N*-acetyl-D-glucosaminidase activity) (Monteiro et al., 2015), we confirm now that BCO affects fungal cells at several levels, and identify metal scavenging as the main mode of action leading to apoptotic cell death. This highly complex and multitarget mode of action, unlikely to promote fungal resistance, combined with its natural origin and the absence of mammal topical toxicity, or genotoxicity may constitute a stepping-stone into a new era of clinical antifungal agents.

## Materials and Methods

### Ethics Statement

All assays involving mammals were conducted in two laboratories with GLP compliance status: Covance Laboratories (England), and Eurofins PSL – Product Safety Labs (United States). At Covance, the following studies were performed in accordance with the requirements of the Animals (Scientific Procedures) Act 1986 and all protocols were previously subjected to the site Ethical Review Process (EPR). For the 21-day study, animal testing was performed under the approval of the Research Ethics Committee (UK Health Departments’ Research Ethics Service, National Health Service, England), with the project license number PPL 70/7602-2. At Eurofins PSL, all acute toxicological studies were performed in accordance with the requirements of 40 CFR Part 160: U.S. EPA (FIFRA), 1989, OECD (as revised in 1997) published in ENV/MC/CHEM (98)17, OECD, Paris, 1998; and EC Directive 2004/10/EC, Official Journal of the European Union, L50/44, Feb. 20, 2004. EUROFINS Product Safety Labs (PSL, United States), is AAALAC accredited, GLP compliant and USDA registered. All protocols of the acute toxicological studies were reviewed and unanimously approved by the Internal Animal Care and Use Committee (IACUC) on the fourteen of May 2010: acute dermal toxicity (PSL protocol #P322), primary eye irritation (PSL protocol #324), dermal sensitization (PSL protocol #P328) and primary skin irritation (PSL protocol #P326).

### Microorganisms and Culture Media

*Saccharomyces cerevisiae* BY4743 and *Candida albicans* CBS 562 were grown in Glucose-Yeast extract-Peptone (GYP) medium (0.5% w/v peptone, 0.5% w/v yeast extract, 2% w/v glucose, and 1.5% w/v agar), for 24 h, at 30 and 34°C, respectively. For the assays described here, the media used were: Synthetic Complete (SC) broth with 2% (w/v) glucose (FORMEDIUM) for *S. cerevisiae*, and Potato-Dextrose-Broth (PDB, DIFCO) buffered at pH 7.5 and Yeast extract-peptone-Dextrose medium (YPD: 2% w/v peptone, 1% w/v yeast extract, and 2% w/v glucose) for *C. albicans.*

### Plant Material

*Lupinus albus* L. seeds were purchased from Inveja SAS (France) and were germinated and grown in growth chambers with a photoperiod of 16 h light/8 h dark at 18°C, for periods up to 10 days. The seed coats were removed and the intact cotyledons were dissected from the axes and stored frozen at -80°C until needed.

### Antifungal Agents

Blad-containing oligomer (BCO) was extracted and purified from 8-days-old cotyledons as previously described (Monteiro et al., 2010) and stored lyophilized at room temperature. Stock solutions of AMB, FLC, and caspofungin (all from SIGMA) were prepared and stored frozen at -20°C until used.

### Global Transcriptome Profiling

The transcriptome profiling of *C. albicans* was analyzed by RNA-sequencing (RNA-seq). Two cultures of *C. albicans* were grown as described below in the ‘Determination of endogenous ROS production’ section, one in the presence of BCO, and the other in its absence (control). After 4 h of incubation, cells were harvested and subjected to a treatment with 0.4 mg/mL lyticase in 50 mM Tris-HCl pH 7.5, 1 M sorbitol, 0.8 M KCl, 10 mM MgSO_4_, and 15 mM β-mercaptoethanol for 1 h at 30°C with gentle stirring to digest the cell wall. Spheroplasts were pelleted by centrifugation and their RNA extracted according to the instructions of the RNA isolation kit (RNeasy Mini Kit – QIAGEN) and stored at -80°C.

Poly(A) mRNA was purified from ca. 25 μg total RNA with two rounds of Dynabeads mRNA DIRECT Micro Purification Kit (Invitrogen). cDNA libraries were constructed with the Ion Total RNA-Seq Kit v2 (Life Technologies) and quantified with Agilent DNA 1000 Kit in the Agilent 2100 Bioanalyzer (Agilent Technologies). The fragments of six barcoded libraries were pooled and clonally amplified by emulsion PCR using the Ion PI Template OT2 200 kit v2 and the Ion OneTouch 2 System (Life Technologies), and the positive Ion Sphere Particles enriched with Ion OneTouch ES (Life Technologies). This procedure was performed twice for a total of two groups of clonally amplified library spheres. Finally, the positive ion spheres were loaded into two Ion PI chip v2 and sequenced in the Ion Proton System (Life Technologies) and Ion PI Sequencing 200 Kit v2 at Genoinseq (Biocant, Cantanhede, Portugal). Ion Proton adapter sequences and low-quality bases were trimmed using the Torrent Suite software (Life Technologies). Duplicate reads were removed using PRINSEQ (Schmieder and Edwards, 2011) and rRNA reads were removed using RiboPicker (Schmieder et al., 2012). The remaining reads were mapped using TMAP version 4.0.6 (Life Technologies) against the reference transcriptome of *C. albicans* (genome *C. albicans* SC5314 A22-s06-m01-r05) (Skrzypek et al., 2016). Read count was performed by eXpress^1^ for each condition (Roberts and Pachter, 2012). Gene name and description for each reference transcript were extracted from the Candida Genome Database annotation files (Skrzypek et al., 2016).

Read counts were uploaded to the Degust^2^ and analyzed using Limma/Voom method, which was incorporated into Degust. Coding sequences with a false discovery rate (FDR) < 0.1 and a fold change greater than 1, were considered significant.

### *S. cerevisiae* HIP HOP Profiling with a Panel of Gene Deletion Signature Strains

In this study we used *S. cerevisiae* as model organism, rather than*C. albicans*, since this is the only yeast species for which a complete deletion mutant strain collection exists (Giaever and Nislow, 2014). BCO was tested on *S. cerevisiae* gene deletion strains, purchased from EUROSCARF (Supplementary Table S1). The 27 selected strains have been previously determined to be characteristic for common modes of action in which a specific target protein is lacking (Hoepfner et al., 2014) and constitute a mini HIP HOP assay (Prescott and Panaretou, 2017). Heterozygous diploids: *nmd3Δ/NMD3, rei1Δ/REI1, lsg1Δ/LSG1, ssl2Δ/SSL2, neo1Δ/NEO1, tim54Δ/TIM54, pik1Δ/PIK1, cmd1Δ/CMD1, tda10Δ/TDA10, mia40Δ/MIA40, tom40Δ/TOM40*, and *mrpl19Δ/MRPL19.* Homozygous diploids: *lem3Δ/lem3Δ, fen1Δ/fen1Δ, lro1Δ/lro1Δ, aft1Δ/aft1Δ, ftr1Δ/ftr1Δ, ctr1Δ/ctr1Δ, fet3Δ/fet3Δ, ire1Δ/ire1Δ, hac1Δ/hac1Δ, sur4Δ/sur4Δ, slt2Δ/slt2Δ, aro1Δ/aro1Δ, trp4Δ/trp4Δ, bck1Δ/bck1Δ*, and *rim101Δ/rim10 Δ*. The diploids have the following isogenic background: *MATa/α; his3Δ1/his3Δ1; leu2Δ0/leu Δ0; met15Δ0/MET15; LYS2/lys2Δ0; ura3Δ0/ura3Δ0* with genes deleted with *KANMX4*. Cultures of each strain were grown to late log phase and their optical density determined with a 1 cm path length spectrophotometer. The cultures were then adjusted to OD_600_ 0.00015 in SC medium and transferred to a transparent polystyrene 384 well microplate. Each microplate well contained 50 μl yeast culture in liquid SC medium with or without BCO. The microplate with lid was then incubated at 30°C in a Tecan Infinite M200 plate reader recording OD_600_ readings in 9 separate locations per well every 20 min between 5 and 27 h. Growth inhibition over 24 h was determined for each strain relative to the corresponding untreated control. The same procedure was carried out for the isogenic control strain BY4743. Each curve is an average of 12 replicate wells.

### Effect of Metallic Ions on BCO Bioactivity

To test the effect of metallic ions on BCO antifungal activity, susceptibility tests were performed on *C. albicans* in the presence of increasing concentrations of these elements. The susceptibility tests were made according to the CLSI – Clinical and Laboratory Standards Institute (former NCCLS – National Committee for Clinical Laboratory Standards) guideline M27-A2 (NCCLS, 2002), with some adjustments, using broth microdilution method, as previously described (Pinheiro et al., 2016). Six different cations were supplied, separately, in the culture medium (PDB): copper (CuCl_2_ and CuSO_4_.5H_2_O), calcium [CaCl_2_.2H_2_O, CaSO_4_ and Ca(NO_3_)_2_], magnesium (MgCl_2_.10H_2_O and MgSO_4_.7H_2_O), iron (FeCl_2_ and FeSO_4_.7H_2_O), zinc (ZnCl_2_ and ZnSO_4_.7H_2_O), and manganese (ZnCl_2_ and ZnSO_4_.7H_2_O). Each cation was tested under different salt forms (described above in round brackets) separately, in order to determine any possible influence of the anion in the results. First, the minimum toxic concentration for each of these cations was determined with concentrations ranging from 4.88 μM to 10 mM. After determining the toxicity lower limit, progressively lower concentrations of each cation were tested with BCO. A culture medium without supplemented cations and with BCO was used as control. All assays were performed in triplicate. The content of each cation in the unsupplemented (original) PDB medium was quantified prior to the addition of the salts. A mixture of three different batches of PDB was homogenized and a single analysis was performed. The mixture was previously acid digested with micro-waves, and the content of the cations was determined by inductively coupled plasma optical emission spectrometry (ICP-OES) (ISO 11885, 2007).

### Quantification of the Plasma Membrane Ergosterol

A fresh culture of *C. albicans* was inoculated in 50 mL YPD medium for 24 h at 34°C, without shaking containing a sub-inhibitory concentration of BCO (2.4 μM). Sub-inhibitory concentrations of FLC (208 μM), AMB (0.27 μM), and caspofungin (26 μM) were also tested as references of ergosterol targeted modes of action (positive control – FLC; negative control – caspofungin; multitarget mode of action – AMB). Cells were harvested by centrifugation and washed with distilled water. Total intracellular sterols were extracted and quantified as previously described (Arthington-Skaggs et al., 1999). Statistical analysis was performed using GraphPad Prism software version 5.02, La Jolla, CA, United States. *P*-values were calculated using one-way ANOVA and Bonferroni multiple comparison post-test.

### Determination of Endogenous ROS Production

A fresh culture of *C. albicans* was inoculated (1 × 10^5^ CFU/mL) in PDB pH 7.5 with 2.4 μM BCO and incubated at 34°C without shaking. After 4 h incubation, intracellular reactive oxygen species (ROS) levels were measured by a fluorometric assay using the fluorescent dye 2,7-dichloro-dihydro-fluorescein diacetate (DCFH-DA; Sigma) as a ROS indicator, as previously described (Favre et al., 2008). PDB pH 7.5 was used as negative control and PDB pH 7.5 with 20 mM H_2_O_2_ (added 30 min before ROS analysis) as positive control.

### Annexin V and PI Staining

Annexin V/propidium iodide (PI) binding assays were performed according to the FITC Annexin V/Dead Cell Apoptosis Kit (Molecular Probes), with minor modifications, as previously described (Du et al., 2008). Two cultures of *C. albicans* were grown as described in the ‘Determination of endogenous ROS production’ section, one in the presence of BCO, and the other in its absence (control).

### Toxicological Studies in Mammals

#### Acute Toxicity

Dermal (according to the guideline OECD 402, U.S. EPA OPPTS 870.1200) (OECD, 1987; United States Environmental Protection Agency, 1998a). (2) Eye irritation (according to the guideline OECD 405, U.S. EPA OPPTS 870.2400) (United States Environmental Protection Agency, 1998b; OECD, 2002b). (3) Skin irritation (according to the guideline OECD 404, U.S. EPA OPPTS 870.2500) (United States Environmental Protection Agency, 1996; OECD, 2002a). (4) Skin sensitisation (according to the guideline OECD 406, U.S. EPA OPPTS 870.2600) (OECD, 1992; United States Environmental Protection Agency, 2003).

#### Short-Term Toxicity

BCO short-term toxicity was investigated in a 22-day topical dermal toxicity study (guideline OECD 410, 1981) (OECD, 1981).

#### *In Vitro* Genotoxicity Testing

(1) Bacterial assay for gene mutation – Ames test (according to the guideline OECD 471, 1997) (OECD, 1997a). In a reverse gene mutation assay in bacteria, *Salmonella typhimurium* histidine-requiring strains TA98, TA100, TA1535, and TA1537 and *Escherichia coli* tryptophan-requiring strain WP2*uvr*A pKM101 were exposed to BCO. Due to the protein nature of the test article, the treat and plate methodology was used in preference to the standard plate-incorporation test, to prevent artifacts due to growth stimulation following the administration of a polypeptide-containing test article. (2) Mammalian assay for gene mutation – mouse lymphoma assay (according to the guideline OECD 476, 1997, also compliant with OECD 490, 28 July 2015) (OECD, 1997b, 2015). (3) Mammalian assay for clastogenicity – p53 competent human lymphocyte assay (according to the guideline OECD 487, 2014) (OECD, 2014a). BCO was tested in an *in vitro* micronucleus assay using duplicate human lymphocyte cultures prepared from blood samples pooled from two male donors in a single experiment.

#### *In Vivo* Studies in Somatic Cells

Comet assay (according to the guideline OECD 489, 2014) (OECD, 2014b). This *in vivo* alkaline single cell gel electrophoresis assay, also called alkaline Comet Assay, is a method to measure DNA strand breaks in eukaryotic cells. Based on their size, DNA fragments migrate in the gel away from the head to the tail, and the intensity of the comet tail relative to the total intensity (head plus tail) reflects the amount of DNA breakage.

### Fluorescence Microscopy

All samples were observed under a fluorescence microscope (Axioscope A1 with phase contrast and epi-fluorescence, Zeiss) equipped with a camera (AxioCam ICm1, Zeiss), using three different filters: Filter Set 49 DAPI (Excitation G 365, Emission BP 420/470); Filter Set 10 FITC/GFP (Excitation BP 450–490, Emission BP 515–565); and Filter Set 15 Rhodamine (Excitation BP 540–552, Emission LP 590).

## Results

### BCO Effects on *C. albicans* Transcriptome and on *S. cerevisiae* Mutants

**FIGURE 1:**
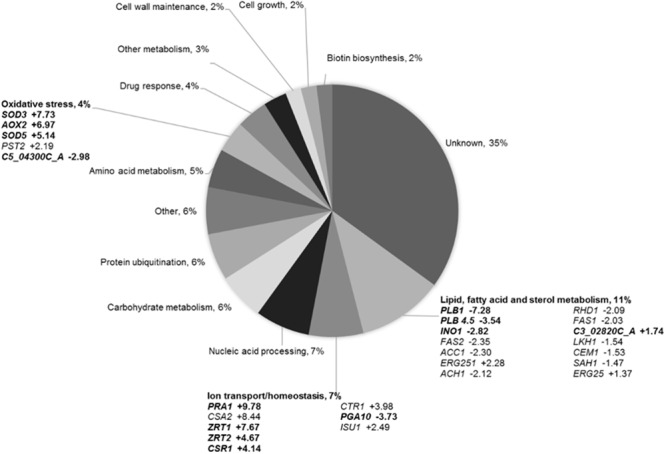
Assigning Blad-containing oligomer (BCO) responsive genes from *Candida albicans* to functional categories. The average logarithmic (log_2_) fold change ratio from three independent experiments is shown. Genes in boldface have a FDR < 0.05. All other genes have a FDR < 0.1.

To analyze BCO genome-wide effects and to identify the mechanisms underlying its fungal growth inhibition (Pinheiro et al., 2016), RNA-seq was employed to analyze *C. albicans* transcriptome upon exposure to BCO for 4 h. A total of 124 genes were found to be differentially expressed. Of these, 80 were up-regulated and 44 were down-regulated comparatively to untreated yeast cultures. The functional distribution of BCO responsive genes is shown in **Figure 1**.

**FIGURE 2:**
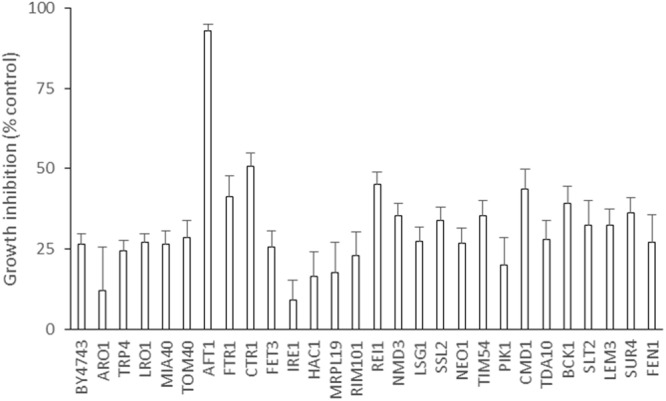
Blad-containing oligomer induced growth inhibition in a panel of yeast HIP HOP “signature strains” that are characteristic for common modes of action. Signature genes were identified previously through high-throughput genome-wide HIP HOP screening of a library of yeast inhibitory small molecules. Growth inhibition of the 27 strains over 24 h was determined in the presence of a sublethal inhibitory dose of BCO (0.024 μM). Cultures of each strain were diluted to OD_600_ 0.00015 and grown in a 384 well plate with and without BCO. Readings were taken every 20 mins for 27 h to produce 48 separate growth curves. Increase in growth over 24 h was then used to determine growth inhibition of each strain relative to the untreated control of the same genetic background. The same procedure was carried out for the isogenic control strain BY4743. Error bars are SEM, *n* = 12.

To confirm some of these results, a yeast chemical genetics approach was carried out using a short list of *S. cerevisiae* mutant strains based on their known hypersensitive profiles. The selected 27 profiles corresponded to 10 common modes of action described for antifungal drugs (Supplementary Table S1). Each yeast deletion strain was screened for hypersensitivity to BCO, as well as the parental strain, using a previously determined (Supplementary Figure S1) sublethal dose of BCO (0.024 μM). The results presented in **Figure 2** clearly show that, under these conditions, the only deletion strain displaying a marked hypersensitivity to the BCO sublethal concentration corresponded to the mutant lacking the transcription factor Aft1, a gene involved on iron utilization and homeostasis (Yamaguchi-lwai et al., 1995).

### Neutralization of BCO Activity by Divalent Cations

Considering the results obtained in the experiments above, namely the hypersensitivity of the yeast mutant strain *aft1Δ/aft1Δ* and the effect on ion transport/homeostasis responsive genes, susceptibility tests to BCO were conducted with *C. albicans* cells in PDB medium supplemented with six divalent cations. The following concentrations of each cation was quantified in the original (unsupplemented) PDB medium: 8 μM Cu^2+^, 18 μM Ca^2+^, 59 μM Mg^2+^, 2 μM Fe^2+^, 3 μM Zn^2+^, and 0.09 μM Mn^2+^. These concentrations are negligible compared to the concentrations added and therefore, only the supplemented concentration was considered for the analysis of the results (**Table 1**). Considering the inherent toxicity of each cation to the cells (**Table 1**), sub-toxic concentrations were tested to assess their individual effect on the activity of BCO against *C. albicans*. As shown in **Table 1**, all cations induced a marked increase in both MIC and MFC values, thus expressing a clear decrease in the antifungal activity of BCO. Ultimately, albeit at different concentrations, all cations were able to neutralize the activity of BCO. The cation potency to neutralize BCO was lower for magnesium, followed by copper, calcium, and iron. Manganese and specially zinc were the cations with the strongest influence on BCO. No differences were found for distinct salts of the same cation nor between replicates.

**Table 1:** Effect of different cations on the Blad-containing oligomer (BCO) growth inhibiting activity upon *Candida albicans* cells in PDB medium.

Cations	Maximum non-toxic cation concentration (μM)^∗^	Concentration of cation supplemented (μM)	MIC of BCO (μM)	MFC of BCO (μM)^∗∗^	Maximum cation concentration with no influence in BCO activity (μM)^∗^
–	–	–	0.19	0.38	–
Cu^2+^	1,250	1,250	>0.76	–	15.62
		625	>0.76	–	
		125	0.76	>0.76	
		62.5	0.38	0.76	
		15.62	0.19	0.38	
Ca^2+^	≥10,000	10,000	>0.76	–	15.62
		1,000	>0.76	–	
		125	0.76	>0.76	
		31.25	0.38	0.76	
		15.62	0.19	0.38	
Mg^2+^	≥10,000	10,000	>0.76	–	125
		1,000	0.38	>0.76	
		500	0.19	>0.76	
		250	0.19	>0.76	
		125	0.19	0.38	
Fe^2+^	1,250	1,250	>0.76	–	15.62
		625	>0.76	–	
		125	>0.76	–	
		62.5	0.76	0.76	
		15.62	0.19	0.38	
Zn^2+^	156	156.2	>0.76	–	3.91
		78.1	0.76	>0.76	
		15.62	0.38	0.76	
		7.81	0.38	0.76	
		3.905	0.19	0.38	
Mn^2+^	625	625	>0.76	–	7.81
		312.5	>0.76	–	
		62.5	0.76	>0.76	
		31.25	0.38	0.76	
		15.62	0.38	0.76	
		7.81	0.19	0.38	

*^∗^Concentration calculated based only on the added amount of each cation. Each cation was tested independently under two or three different salt formulations (Cu^*2+*^: CuCl_*2*_/CuSO_*4*_.5H_*2*_O; Ca^*2+*^: CaCl_*2*_.2H_*2*_O/CaSO_*4*_/Ca(NO_*3*_)_*2*_; Mg^*2+*^: MgCl_*2*_.10H_*2*_O/MgSO_*4*_.7H_*2*_O; Fe^*2+*^: FeCl_*2*_/FeSO_*4*_.7H_*2*_O; Zn^*2+*^: ZnCl_*2*_/ZnSO_*4*_.7H_*2*_O, and Mn^*2+*^: MnCl_*2*_.4H_*2*_O/MnSO_*4*_). There was no variation among triplicates of the MIC and MFC tests.*

*^∗∗^ (-) MFC was not determined because there was no well without growth.*

### BCO Interferes with the Yeast Cell Membrane Ergosterol Content

**FIGURE 3:**
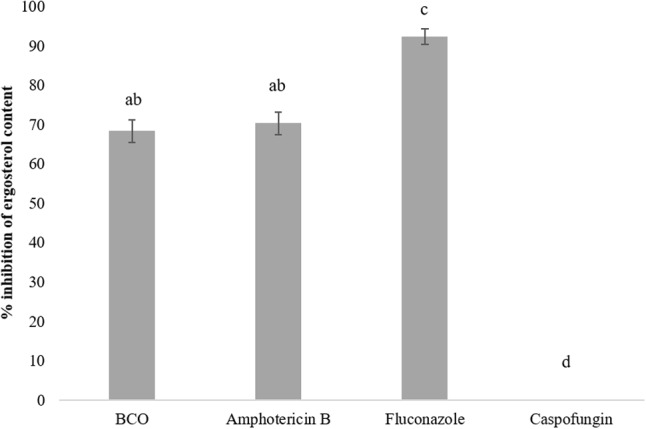
Effect of sub-inhibitory concentrations of BCO, amphotericin B (AMB), FCL, and caspofungin on the inhibition of ergosterol synthesis in *C. albicans*. The results represent the mean ± standard deviation of three independent experiments. Statistical analysis was performed as described in the “Materials and Methods” section. Bars with a letter in common are not significantly different (*P* > 0.05).

The set of genes in the category of lipid, fatty acid, and sterol metabolism was among the most responsive in the RNA-seq experiment (**Figure 1**) and, therefore, the effect of BCO on the ergosterol content of *C. albicans* plasma membrane was assessed. As a positive control, total ergosterol content was also determined after exposing the cells to FLC. AMB was used as a reference control for a cell membrane disruption mode of action without direct interference in the biosynthesis of ergosterol. Caspofungin was used as negative control. The results (**Figure 3**) show that *C. albicans* suffered a 91% reduction in its ergosterol contact when exposed to FLC, as compared to the control. This reduction was significantly lower when the culture was exposed to AMB (71%) or BCO (69%) whereas caspofungin did not interfere with the ergosterol content at all. No statistical differences were observed in the reduction of the ergosterol content between AMB and BCO.

### Induction of *C. albicans* Apoptosis by BCO

**FIGURE 4:**
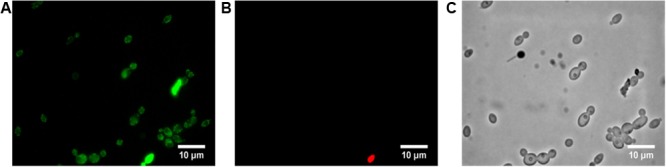
Induction of *C. albicans* apoptosis by BCO. Annexin V and propidium iodide staining of *C. albicans* cells exposed to 2.4 μM BCO for 4 h. Cells labeled with annexin V **(A)**, propidium iodide **(B)**, or simply observed by bright field microscopy **(C)**. Bar corresponds to 10 μm.

The third interesting set of responsive genes obtained with the RNA-seq study was the oxidative stress associated genes (**Figure 1**). This gene family has long been associated to cell apoptosis and previous studies have already shown the induction of cell death by BCO (Pinheiro et al., 2016). To investigate whether BCO lethal effect occurs via induction of apoptosis, and/or necrosis, annexin V and PI staining were performed (**Figure 4**). An early marker of apoptosis is the exposure of phosphatidylserine (PS) on the cell surface, since it is normally located in the luminal layer of the cytoplasmic membrane (Martin et al., 1995; Madeo et al., 1997). Annexin V displays a high affinity for PS and its previous labeling with fluorescein isothiocyanate (FITC) allows the visual identification of apoptotic cells under a fluorescence microscope. Staining with PI allows detection of damaged membranes. Therefore, differential staining patterns discriminate among live cells (annexin V-/PI-), early apoptosis (annexin V+/PI-), necrosis (annexin V-/PI+), and late apoptosis/necrosis (annexin V+/PI+) (Hao et al., 2013). The majority of *C. albicans* cells exposed for 4 h to 2.4 μM BCO were only green in color, which is indicative of early apoptosis (**Figure 4A**). Some cells were stained with both dyes, meaning that, at this point, a minority of cells was undergoing late apoptosis (**Figures 4A,B**). Cells grown in the absence of BCO were not stained by annexin V or PI (data not shown).

### ROS Are Produced during BCO Induced Apoptosis

**FIGURE 5:**
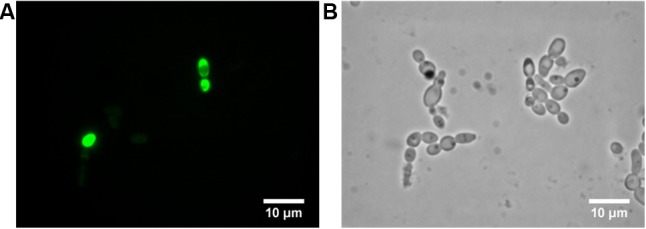
Detection of endogenous reactive oxygen species (ROS) production. DCFH-CA staining of *C. albicans* cells exposed for 4 h to 2.4 μM BCO. Cells labeled with DCFH-DA **(A)** or simply observed by bright field microscopy **(B)**. Bar corresponds to 10 μm.

Production of reactive oxidative species (ROS) by *C. albicans* exposed for 4 h to 2.4 μM BCO was analyzed with DCFH-DA, a non-fluorescent ROS indicator which diffuses across the cell membrane, undergoes oxidation and emits green fluorescence (**Figure 5**). Appropriate controls were cells grown in the absence of BCO (negative control) and cells exposed to 20 mM H_2_O_2_ for 30 min (positive control). After 4 h of incubation with BCO, ROS accumulation was already detectable in some cells (**Figure 5A**). The negative control did not present DCFH-DA staining, whereas in the positive control 100% cells were stained (data not shown).

### Absence of Topical Toxicity (Acute and Short Term) and Genotoxicity upon Exposure of Mammals to BCO (*In Vitro* and *In Vivo* Assays)

#### Acute Toxicity

No gross signs of clinical toxicity, adverse pharmacologic effects or abnormal behavior were observed following dermal (10 rats), eye irritation (3 New Zealand White rabbits), skin irritation (3 New Zealand White rabbits), and skin sensitization (20 Guinea pigs). There were also no signs of toxicity observed at necropsy in all tests. A summary of the overall results for BCO acute toxicity is shown in **Table 2**.

**Table 2:** Summary of acute toxicity of BCO.

Type of study	Species	Result
Dermal	Rat	LD_50_: 400 mg BCO/kg
Skin irritation	Rabbit	Slightly irritating
Eye irritation	Rabbit	Mildly irritating
Skin sensitization	Guinea pig	Non-sensitizing

*LD_*50*_ – median lethal dose.*

Blad-containing oligomer gave rise to non-toxic classification resulting from rat dermal (LD_50_ 400 mg BCO/kg). Although the skin and eye irritation studies showed a slightly or mildly irritating effect, these results were insufficient to warrant classification according to Annex I of Regulation (EC) No. 1272/2008 (of the European Parliament and of the Council of 16 December 2008 on the classification, labeling and packaging of substances and mixtures). Furthermore, BCO was not a skin sensitiser in guinea pig, using the Buehler method.

### Short-Term Toxicity

In the 22-day dermal test, no clinical observations of toxicological importance, no deaths and no clinical signs of toxicity at necropsy were noted in all groups of rats tested [Crl:WI (Han) rats, 5 sex^-1^ dose^-1^].

#### Genotoxicity Testing

These results are summarized in **Table 3**.

**Table 3:** Summary of genotoxicity testing of BCO.

Type of study	Test system	Dose range tested^a^	Result^a^
*In vitro*	Bacterial (5 strains,Ames test, treat and plate methodology)	+/-S9: 3.2–1000 μg/plate	±S9: negative
*In vitro*	Mammalian (L5178Y *TK*^+/-^) gene mutation	Experience 1: 3h –S9: 100–400^b^ μg/mL 3h +S9: 30–180^c^ μg/mL Experience 2: 3h –S9: 100–500 μg/mL 3h +S9: 60–240 μg/mL	–S9: negative +S9: positive
*In vitro*	Mammalian (cultured human lymphocytes) micronucleus	3 (+21) h +/-S9: 0–400 μg/mL 24 (+24) h –S9: 20–320^c^ μg/mL	±S9: negative
*In vivo*	Rat stomach comet	0, 100, 200, 400 mg/kg bw/day	Negative

*^a^ S9 from rat liver.*

*^b^ Higher concentration tested without precipitate at the end of treatment.*

*^c^ Concentration limited by toxicity.*

#### *In Vitro* Genotoxicity Testing

Bacterial assay for gene mutation: No evidence for BCO induced mutagenic activity was detected in the system tested (three *S. typhimurium* histidine-requiring strains, and one *E. coli* tryptophan-requiring strain) either in the presence nor in the absence of a rat liver metabolic activation system (S9). Mammalian assay for gene mutation: In the absence of the activation system S9, BCO did not induce gene mutation. In the presence of S9, there was an increase in mutant frequency (MF) that exceeded the sum of the Global Evaluation Factor (GEF) + negative control for the highest concentrations tested. In those cases, increases in both small (predominantly) and large colony mutant frequencies were observed. Under the conditions of this study the data constituted a positive result in the presence of S9 and indicated the possibility that the increase in MF observed is potentially being driven by a clastogenic type of mechanism. However, the clastogenicity assay performed in mammalian cells (with cultured human lymphocytes) was negative.

#### *In Vivo* Genotoxicity Testing

Comet assay. There was no dose-related increase in % hedgehogs in stomach following treatment with BCO, thus demonstrating that treatment with BCO did not cause excessive DNA damage (which can interfere with Comet analysis) following oral gavage administration, nor did mechanical/enzyme-induced damage result during sample preparation. BCO did not originate an increase in the incidence of DNA damage, as measured by % tail intensity in the stomach of male rats (20/dose), following oral dosing up to a level of 400 mg kg^-1^ bw day^-1^.

## Discussion

The limited repertoire of antifungal drugs to treat human fungal infections, further restrained by increasing events of host toxicity and drug resistance, has created an urgent and desperate need for new drugs with novel mechanisms, for both agricultural and clinical applications. The most up-to-date research for antifungal drug discovery is focused in alternative treatments with drugs aimed at novel fungal targets within the cellular circuitry crucial for stress response (Lamoth et al., 2014), drug resistance (Holmes et al., 2016), and virulence (Vila et al., 2017), although the classical research for novel targets aimed at cell survival still prevails (Balouiri et al., 2016). Here we describe a novel mode of action of a new, natural and multitarget antifungal compound (BCO) and assess its toxicity toward mammalian cells.

In a genome-wide analysis of the transcriptome from *C. albicans* exposed to BCO, the most representative functional class of responsive genes included lipid, fatty acid, and sterol metabolism: three genes involved in sterol biosynthesis (*C3_02820C_A, ERG25*, and *ERG251*) were up-regulated, suggesting a response to ergosterol depletion (Liu et al., 2005), whereas genes belonging to the lipid (*PLB1, INO1, PLB4.5, RHD1, LKH1*, and *SAH1*) and fatty acid metabolisms (*ACH1, FAS2, CEM1, ACC1*, and *FAS1*) were all down-regulated. Downregulation of these genes has already been described in *C. albicans* after AMB exposure (Liu et al., 2005), and suggests a disruptive effect of BCO at the cell membrane level (Spampinato and Leonardi, 2013). Ion transport/homeostasis seems to play a key role on BCO bioactivity upon *C. albicans* cells, as the expression of genes belonging to this class were also largely affected, particularly those related to zinc homeostasis: upregulation of *CSR1, ZRT1, ZRT2*, and *PRA1* (Citiulo et al., 2012; Böttcher et al., 2015). The over-expression of these genes is induced by mild zinc deficiency (Wu et al., 2008) originated by its putative sequestration. Zinc is, after magnesium, the second most widespread metal present in enzymes belonging to all six major functional classes (Andreini et al., 2008), and zinc ions are essential for a wide variety of biochemical processes. Control of zinc homeostasis is especially relevant for pathogens because the amount of labile zinc in host tissues is very low. Accordingly, mammals have developed the capacity to inhibit microbial growth in their tissues by zinc starvation, as part of a broader defense mechanism termed “nutritional immunity” (Hood and Skaar, 2012). Besides zinc, both copper and iron are also required to organisms, primarily through their role as cofactors in essential metabolic functions (Marvin et al., 2003; Lan et al., 2004). They were both also affected by BCO: *CSA2*, a gene involved in a heme-iron uptake system in *C. albicans* (Weissman and Kornitzer, 2004) was up-regulated, which is in accordance with a low-iron environment, and *CTR1*, a copper ion transporter, was also up-regulated, probably due to its essential role in iron uptake (Lan et al., 2004; Lee et al., 2005). BCO also seems to impose oxidative stress inside yeast cells, as upregulation was also observed on four oxidative stress-related genes: *SOD3* and *SOD5, AOX2*, and *PST2* (Liu et al., 2005; Frohner et al., 2009; Li et al., 2015). This was corroborated by downregulation of *C5-04300C_A* gene, a homolog of *S. cerevisiae DUG1* gene, which encodes a glutamine amidotransferase involved in glutathione catabolism (Toledano et al., 2013), given that glutathione acts as an important line of defense against ROS in bio-reductive reactions (Mendoza-Cózatil et al., 2005). Genes involved in biotin biosynthesis, namely *BIO2, BIO3*, and *VHT1* (Chattopadhyay et al., 2009) were also down-regulated upon exposure to BCO. This may derive from downregulation of genes involved in lipid and fatty acid metabolisms, because phospholipid precursors play an essential role in biotin biosynthesis (Santiago and Mamoun, 2003). In addition, biotin synthesis is also repressed under low-iron conditions (Shakoury-Elizeh et al., 2004). Either way, this repression in biotin synthesis is most likely a secondary effect of the exposure to BCO rather than one of its primary targets. In the same segment are the genes related to nucleic acid processing, carbohydrate metabolism, protein ubiquitylation, amino acid metabolism, drug response, and cell growth.

The effect of metal ions on BCO activity was further elucidated in *S. cerevisiae*, using HIP HOP profiling with a panel of gene deletion signature strains. From all the deletion mutant strains tested, only the homozygous deletion strain *aft1Δ/aft1Δ* displayed a marked sensitivity to BCO. *AFT1* is a transcription factor involved in iron utilization and homeostasis (Yamaguchi-lwai et al., 1995). These results rule out the hypothesis of a direct effect of metals on the activity or structure of BCO, and suggest that interference with iron homeostasis and/or other iron metabolic reactions are involved in BCO mode of action. Previous results have already demonstrated the metal-binding activity of BCO *in vitro* (Supplementary Table S2). It was demonstrated that in the presence of metallic ions the melting temperature of BCO shifts in several degrees allowing to conclude that they are, potentially, strong binding ligands for BCO. Furthermore, a broader BCO cation scavenging activity was observed in susceptibility tests against *C. albicans*, with a complete neutralization of its antifungal activity by several divalent cations (zinc, manganese, iron, copper, and calcium).

The alterations in gene expression related to cell membrane and ergosterol, as well as the previously described destabilizing effect of BCO on yeast cell membranes (Pinheiro et al., 2016), could indicate an interference of BCO with ergosterol synthesis. This isoprenoid is the main sterol found in fungal and protozoa cell membranes, and is also the target of many well-known drugs, including azoles. However, our results seem to indicate that BCO destabilizes the cell membrane, but not in an ergosterol metabolic pathway-dependent way. Similarly to other antifungals (Munoz et al., 2006; Van Der Weerden et al., 2008), this seems to be a secondary target of BCO, clearly undermining cell stability, but without an evident effect on cell growth and/or viability *per se*.

The externalization of PS here demonstrated, combined with the detection of endogenous ROS production and the upregulation of oxidative stress response genes upon exposure of *C. albicans* to BCO, are consistent with an apoptotic cell death. The same mechanism has already been described for AMB (Phillips et al., 2003) and for other antifungals (Wu et al., 2010; Aerts et al., 2011; Emrick et al., 2013).

Blad-containing oligomer was also thoroughly evaluated in a range of genotoxicity assays performed both *in vitro* and *in vivo*. Concerns raised over the increase in MF in the mouse lymphoma assay were not replicated *in vitro* in either the Ames study (from a gene mutation mode of action pathway) or in the p53 competent human lymphocyte assay (from a structural chromosomal mode of action pathway). Furthermore, the *in vivo* rat stomach comet was concluded to be devoid of any DNA damage. Overall, it may be concluded from the evidence presented here that BCO is devoid of any genotoxic potential. No carcinogenicity studies have been conducted because there is no evidence in the available literature to suggest that proteins similar to BCO, comprising polypeptide segments of β-conglutin are associated to an increased incidence of cancer. On the contrary, many legume seed proteins have been claimed to exhibit anticancer potential (Quiroga et al., 2015). Therefore, BCO is unlikely to be considered a carcinogen.

In summary, we demonstrate here that BCO has a disruptive effect on the cell membrane, allowing its entry into the cell, but which is not ergosterol-dependent. Similarly to its known interference with the cell wall (Pinheiro et al., 2016), this does not seem to be its primary antifungal mode of action. The available evidence suggests that BCO primary mode of action is a metal scavenging activity, with a particular effect on zinc and iron, shattering cell homeostasis. This activity might be exerted both extra- and intracellularly. An oxidative stress is then generated inside the cells, culminating in an apoptotic cell death. BCO showed no evidence of topical toxicity toward mammalian cells after acute or short-term expositions. The absence of topical toxicity, genotoxicity, and carcinogenicity of BCO in mammals is of outmost importance to enable its potential application to treat topical fungal infections. Moreover, the metal homeostasis at the host-pathogen interface, which is BCO’s primary mode of action, is now recognized as a preferential target for the development of new antifungal drugs, because metal chelators seem to be beyond drug resistance (Polvi et al., 2016). Further studies are now required to fully understand its interaction with fungal cells (namely, its mode of cell entrance), and with the human body (toxicity and stability after intravenous administration) in order to determine its potential for the treatment of systemic fungal infections. Nevertheless, BCO is already an undoubtedly powerful, novel and very wide-spectrum tool exhibiting multitarget modes of action, and with an apparently very low risk for fungal resistance.

## Author Contributions

SM and AC conceived the project and designed experiments. AP and AC performed the RNA-sequencing experiments. TP performed the yeast chemical genetic experiments. AP performed the studies on metal ions, ergosterol content, endogenous ROS productions and apoptosis studies. AC analyzed and compiled the toxicological studies. AP, AC, SM, and TP wrote the paper and RF approved the final version to be published.

## Conflict of Interest Statement

The authors declare that the research was conducted in the absence of any commercial or financial relationships that could be construed as a potential conflict of interest. The handling Editor declared a shared affiliation, though no other collaboration, with two of the authors and states that the process nevertheless met the standards of a fair and objective review.

## Footnotes

1 https://github.com/adarob/eXpress/

2 http://www.vicbioinformatics.com/degust/
