# Supplementary material for: Bridging the Gap to Non-toxic Fungal Control: Lupinus-Derived Blad-Containing Oligomer as a Novel Candidate to Combat Human Pathogenic Fungi
Source: Front Microbiol. 2017 Jun 28;8:1182. doi: 10.3389/fmicb.2017.01182 (PMC5487463; doi:10.3389/fmicb.2017.01182)
Supplement: Supplementary file 1 [file Data_Sheet_1.docx]

***Supplementary Material***

Bridging the gap to nontoxic fungal control: Lupinus-derived Blad-Containing Oligomer as a novel candidate to combat human pathogenic fungi

Ana Margarida Pinheiro^1^, Alexandra Carreira^2^, Thomas A.K. Prescott^3^, Ricardo B. Ferreira^1^, Sara Monteiro^1,2,*^

^1^LEAF – Linking Landscape, Environment, Agriculture and Food

Instituto Superior de Agronomia, Universidade de Lisboa, 1349-017 Lisboa, Portugal

^2^CEV, SA, Parque Industrial de Cantanhede/Biocant-Park, lote 120, 3060-197 Cantanhede, Portugal

^3^Royal Botanic Gardens, Kew, Richmond, Surrey TW9 3AB, UK

^*^CORRESPONDING AUTHOR:

Sara Alexandra Monteiro; telephone: 351.919013796; fax: 351.213653195; e-mail: sam@cev.com.pt


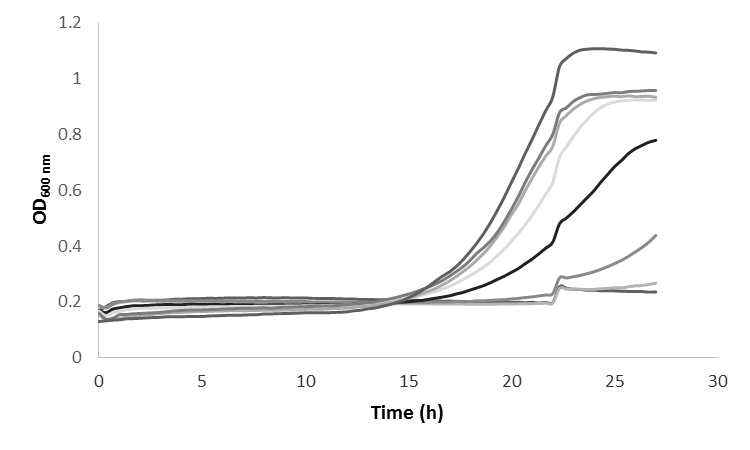


**C**

**E**

**G**

**A**

**D**

**F**

**H**

**B**

**Supplementary Figure S1. Growth curves of S. cerevisiae BY4743 parental strain in SC medium supplemented with 2% (w/v) glucose.** Each growth curve is the average of 12 replicate wells. The OD _600 nm_ of each individual well is an average of nine readings made at different locations of the well. The top line (**A**) refers to the untreated culture (absence of BCO) and the other lines represent the growing culture in the presence of increasing BCO concentrations: 0.012 µM, 0.024 µM, 0.047 µM, 0.095 µM, 0.19 µM, 0.38 µM and 0.76 µM (**B** to **H**, respectively).

**Supplementary Table S1.** ***S. cerevisiae* deletion strains obtained from Euroscarf**. The strains marked HIP (haploinsufficiency profiling) are deleted for one copy of the gene in question. Those marked HOP (homozygous profiling) are deleted for both copies of the gene.

| **Gene** | **Systematic**  **Name** | **Accession Number** | **HIP or HOP** | **Likely mode of action** |
| --- | --- | --- | --- | --- |
| ***NMD3*** | YHR170W | Y26418 | HIP | DNA intercalation |
| ***REI1*** | YBR267W | Y23407 | HIP |  |
| ***LSG1*** | YGL099W | Y24466 | HIP |  |
| ***SSL2*** | YIL143C | Y22302 | HIP |  |
| ***NEO1*** | YIL048W | Y21441 | HIP | Membrane perturbation |
| ***TIM54*** | YJL054W | Y21369 | HIP |  |
| ***PIK1*** | YNL267W | Y26958 | HIP |  |
| ***CMD1*** | YBR109C | Y23248 | HIP |  |
| ***TDA10*** | YGR205W | Y24835 | HIP |  |
| ***BCK1*** | YJL095W | Y31328 | HOP |  |
| ***SLT2*** | YHR030C | Y30993 | HOP |  |
| ***LEM3*** | YNL323W | Y31121 | HOP |  |
| ***SUR4*** | YLR372W | Y35281 | HOP | Membrane perturbation/lipids |
| ***FEN1*** | YCR034W | Y35763 | HOP |  |
| ***ARO1*** | YDR127W | Y34061 | HOP | Plasma membrane stress, internalization of amino acid permeases |
| ***TRP4*** | YDR354W | Y34191 | HOP |  |
| ***LRO1*** | YNR008W | Y35383 | HOP | Fatty acid disruption |
| ***MIA40*** | YKL195W | Y27033 | HIP | Mitochondrial membrane perturbation |
| ***TOM40*** | YMR203W | Y20789 | HIP |  |
| ***AFT1*** | YGL071W | Y34438 | HOP | Cation chelation |
| ***FTR1*** | YER145C | Y36142 | HOP |  |
| ***CTR1*** | YPR124W | Y35539 | HOP |  |
| ***FET3*** | YMR058W | Y36192 | HOP |  |
| ***IRE1*** | YHR079C | Y31907 | HOP | ER stress |
| ***HAC1*** | YFL031W | Y35650 | HOP |  |
| ***MRPL19*** | YNL185C | Y22027 | HIP | Uncoupling |
| ***RIM101*** | YHL027W | Y30936 | HOP | pH stress |

**Supplementary Table S2. Melting temperatures of BCO in the presence of 10 mM of different metallic ions**

| Additives | Melting temperature (°C) |
| --- | --- |
| Control (H_2_O) | 68.5; 73.5 |
| MgCl_2_ | 83 |
| CaCl_2_ | 85 |
| MnCl_2_ | 86 |
| ZnCl_2_ | 62.5; 82.5 |
| ZnSO_4_ | 64.5; 82.5 |
| NiCl_2_ | 86 |

The results in Supplementary Table S2 were obtained by Thermal shift (Thermofluor) assay using BCO at 0.25 µg/µL, as described in (Boivin et al., 2013). When in water, BCO presents two melting temperatures, which corresponds to two different unfolded stages. This is probably due its nature as an oligomer and may indicate multiple denaturation components.. The addition of the divalent metallic ions had a stabilizing effect on the BCO, considering the significant increased on its melting temperature. This means that the BCO-metallic ions binding stabilized the oligomer by minimizing its aggregation and increasing the temperature of denaturation.

Boivin, S., Kozak, S., and Meijers, R. (2013). Optimization of protein purification and characterization using Thermofluor screens. *Protein Expr. Purif.* 91, 192–206. doi:10.1016/j.pep.2013.08.002.
